# Bortezomib combined with lenalidomide as the first-line treatment for the rare synchronous occurrence of multiple myeloma and pulmonary adenocarcinoma: A case report

**DOI:** 10.1097/MD.0000000000005787

**Published:** 2017-01-10

**Authors:** Wenli Zuo, Xinghu Zhu, Jingke Yang, Zhenyang Mei, Mei Deng, Quande Lin, Yongping Song, Qingsong Yin

**Affiliations:** Department of Hematology, Affiliated Cancer Hospital of Zhengzhou University, Zhengzhou, Henan, China.

**Keywords:** bortezomib, case report, lenalidomide, multiple myeloma, pulmonary adenocarcinoma, synchronous

## Abstract

**Background:**

Simultaneous multiple myeloma (MM) and pulmonary adenocarcinoma is a rare occurrence, and thus, treatment is a challenge. This study reports on 1 such case of MM with concurrent lung cancer, where an accurate diagnosis was made and the patient underwent treatment for both cancers.

**Case summary:**

A 68-year-old man presented with 2 months of progressive lower back pain. Visualization with magnetic resonance imaging (MRI) revealed multiple collapsed vertebrae from T12 to S3, as well as an altered signal intensity at the T3 vertebra. The patient was diagnosed with MM upon examination. A chest computed tomography (CT) scan revealed a round mass in the left lower lobe of the lungs, and a CT-guided needle biopsy uncovered a moderately differentiated adenocarcinoma. There were no additional notable findings in the left lung using positron emission tomography computed tomography (PET-CT). Therefore, a diagnosis of MM with pulmonary adenocarcinoma was made. Surgery was performed to excise the lung cancer. Bortezomib was used as first-line induction therapy against both tumors and lenalidomide was used for maintenance. The patient went into complete remission. Using this combined chemotherapy, the patient has survived for over 3 years since a diagnosis was made despite relapsing twice after the first year.

**Conclusion:**

This report clearly delineates the diagnosis and treatment of a rare case of synchronous MM and pulmonary adenocarcinoma, as well as depicts a potentially positive outcome for the patient. It also overviews some diagnostic and therapeutic implications for clinicians.

## 1 Introduction

Multiple myeloma (MM) is a neoplasm of B cells characterized by excessive proliferation of abnormal plasma cells. These malignant plasma cells produce immunoglobulins that are detectable in the serum and/or urine of affected patients.^[1]^ A diagnosis of a secondary solid neoplasm in patients with MM is uncommon, and the pathogenesis underlying this is unknown. It has been suggested that MM patients may have immunological tolerance for developing solid tumors due to an impaired immune system.^[2]^ Synchronous occurrence of MM and lung cancer in a patient is a rare occurrence. Upon performing a literature search on PubMed, only 5 such cases were found^[2–6]^ with only 1 of these cases including a pulmonary adenocarcinoma. Due to this rareness, treatment is not standardized, and, therefore, patient prognosis remains poor.^[5]^ This report covers the clinical features and treatment for a case of synchronous MM and pulmonary adenocarcinoma. Following treatment, both cancers have remained stable, and the patient continues to receive medical follow-up.

## 2 Case presentation

**Figure 1:**
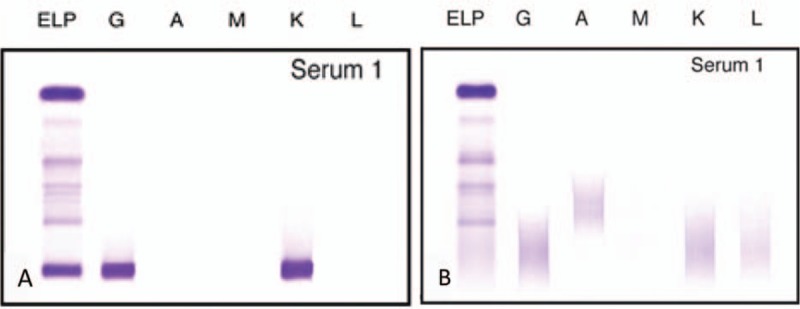
(A) Pretreatment, an immunoglobin G monoclonal band containing a kappa light chain was visualized using immunoelectrophoresis (IEP) analysis. (B) Post-treatment, the monoclonal band had disappeared. Serum 1 refers to the patient's serum. IEP = immunoelectrophoresis.

**Figure 2:**
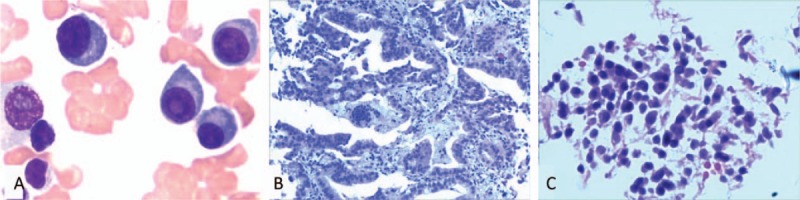
(A) There was an increased population of abnormal plasma cells with dysplastic features in the bone marrow (Wright staining ×1000). (B) A CT-guided needle biopsy of the left lung sampled moderately-differentiated adenocarcinoma (H&E ×600). (C) Pathological analysis of the T3 tissue found diffuse infiltration of plasma cells with unclear intranuclear findings and unevenly distributed nuclei (H&E ×600). CT= computed tomography, H&E = hematoxylin and eosin staining.

**Figure 3:**
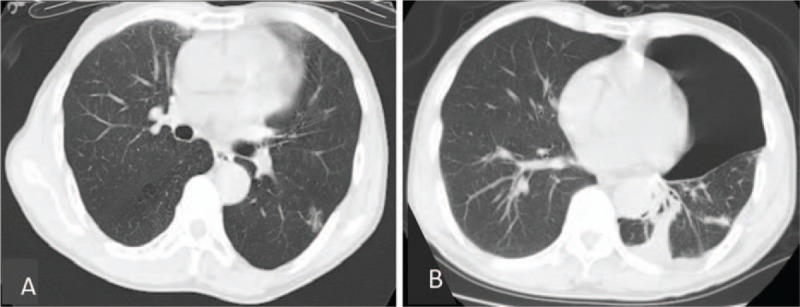
(A) Before treatment, CT-scans revealed a round mass with ground glass opacities in the lower left lobe of the lungs. (B) After surgery, the CT-scan displayed the shadow of the left lower lung wedge resection. CT= computed tomography.

A 68-year-old man was admitted to the Department of Hematology at the Affiliated Cancer Hospital of Zhengzhou University in February 2013 presenting with 2 months of progressive lower backache. With the exception of his back pain, he was otherwise in good health and he had no documented history of disease. However, he had a history of smoking 40 packs of cigarettes per year. The physical examination revealed the pain was located in the back and extended to the waist, and there was slight decrease in breath sound on the left side of the chest. Magnetic resonance imaging (MRI) revealed the collapse of multiple vertebrae from T12 to S3, as well as an altered signal intensity in the T3 vertebra. Laboratory test results included a hemoglobin level of 10.7 g/dL, an elevated total serum protein level of 96 g/L, a reversed A/G ratio of 0.6, a serum creatinine level of 140 μmol/L, a serum urea nitrogen level of 11.3 mmol/L, and a serum beta-2 microglobulin level of 8.55 mg/dL. In addition, he had a serum immunoglobulin G (IgG) of 40.76 g/L, a kappa light chain value of 8.65 g/L and a k/λ of 27.90. Analysis using serum electrophoresis and immunoelectrophoresis (IEP) revealed a monoclonal band that was IgG with a kappa light chain (Fig. 1A). Other serum biochemistry parameters were within normal range. Bone marrow (BM) aspirate had an increased population of abnormal, dysplastic plasma cells (23%) (Fig. 2A). Chest CT scan uncovered a round mass with ground glass opacities in the left lower lobe of the lungs (Fig. 3A). A CT-guided needle biopsy of the left lung revealed a moderately differentiated adenocarcinoma (Fig. 2B), whereas a plasmacytoma was discovered in the T3 tissue. This plasmacytoma consisted of atypical plasma cells with irregular nuclei and cytoplasmic vacuolation, as assessed by haematoxylin and eosin (H&E) staining (Fig. 2C). Immunostaining demonstrated a predominance of CD138+ cells (data not shown). Aside from the findings involving the left lung and multiple vertebrae, no other positive findings were identified on PET-CT scan. Based on the patient evaluation described above, a diagnosis of Durie-Salmon stage IIB/ISS stage III MM was made with co-occurring stage I moderately differentiated lung adenocarcinoma.

The patient was given cyclophosphamide (200 mg/m^2^, days 1–5) and dexamethasone (10 mg, days 1–5) to relieve the symptoms of the lower backache. Ten days post-diagnosis, a left lower lobectomy, as well as hilar and mediastinal lymph node dissection, was performed using video-assisted thoracic surgery (VATS) (Fig. 3B). Chemotherapy was then started in April 2013, consisting of 4 cycles of bortezomib (1.0 mg/m^2^ given on days 1, 4, 8, and 11 from start of treatment) and dexamethasone (20 mg give on days 1, 2, 4, 5, 8, 9, 11, and 12 from start of treatment). The patient experienced no severe complications during the 3 cycles of chemotherapy with the exception of mild peripheral neuritis. During the fourth cycle, the patient suffered from bronchopneumonia that clinically manifested as a fever and a cough with yellow sputum. A sputum culture and drug sensitivity test identified vancomycin-sensitive *Staphylococcus aureus* as the infecting agent. Chemotherapy was continued concurrently with vancomycin given to treat the infection. However, the dose of dexamethasone was reduced to 10 mg from the original 20 mg. The patient achieved complete remission (CR) by the end of the 4 cycles of chemotherapy (Fig. 1B). Home maintenance therapy of lenalidomide (10 mg/day, days 1–28 from start of treatment) was started in November 2013. The patient was seen for regular follow-up and was in CR until August 2014 when a repeat chest CT scan revealed a recurrence of the lung cancer. In addition, IEP analysis found that the IgG and kappa light chain levels were once again elevated. Because the patient could not financially afford bortezomib, treatment was changed to gemcitabine (1.0 g/m^2^ given on days 1 and 8 from start of treatment) with cisplatin (20 mg/m^2^ given on days 1–4 from start of treatment) and dexamethasone (10 mg given on days 1–4 from start of treatment). After 4 cycles, the patient achieved partial remission (PR) and was maintained with lenalidomide at home. The patient presented again at the hospital in November 2015 with chest tightness and shortness of breath. A repeat chest CT scan revealed another relapse of the lung cancer with pleural effusion. Therapy was initiated and consisted of 4 cycles of vinorelbine (25 mg/m^2^ given on days 1 and 8 from start of treatment) and cisplatin (20 mg/m^2^ given on days 1–4 from start of treatment). PR was again achieved, and the patient was again maintained on lenalidomide at home. Through April 2016, the patient continued with regular follow-ups, and both cancers have remained stable and have not progressed.

## 3 Discussion

MM is characterized by neoplastic proliferation of a single clone of monoclonal plasma cells. Secondary neoplasms in MM patients typically occur after radiotherapy or chemotherapy. The presence of a second primary tumor is rare and can easily be missed or misdiagnosed. Specifically, a patient presenting with MM in conjunction with primary lung adenocarcinoma is very rare with only 1 published case, which resulted in a poor prognosis.^[5]^ However, some studies have found an association between MM and an increased risk of secondary malignancy, including hematologic malignancies and solid cancers.^[7–9]^ Currently, the pathogenesis behind the occurrence of secondary cancers in MM patients is unknown and may be complicated by biological factors, familial or genetic predisposition, endocrine factors, and immune dysfunction. It is unclear whether there are separate factors that facilitate the occurrence of a second solid tumor or the same pathogenic factors implicated in MM pathogenesis contribute to the development of a second tumor.^[10,11]^

MM often presents with nonspecific symptoms. When MM occurs concurrently with another solid tumor, MM is typically diagnosed during follow-up and comprehensive inspections of patients with known solid tumors. However, in the case presented here, the presenting symptom was bone pain, and MM was the initial diagnosis following examinations of BM, EP, IEP, and so on. During a comprehensive diagnostic work-up, a mass was found in the left lung using CT. Because extramedullary infiltration occurs in some cases of MM, a CT-guided needle biopsy was performed to assess the nature of the mass in the left lung and the soft tissue mass in the T3 vertebra. A PET-CT scan was also performed to aid in assessing tumor stage and clinical decision-making. After extensive work-up, the patient was diagnosed with Durie–Salmon stage IIB/ISS stage III MM and stage I moderately differentiated lung adenocarcinoma.

Due to the extreme rarity of cases similar to the one described in this article, there is not a standardized treatment. In the sole previously reported case, treatment was directed at the solid tumor due to its more advanced state, and the resulting prognosis was associated with this primary cancer. Some scholars have postulated that a primary malignancy of the hematopoietic system may result in an even worse prognosis than a primary solid tumor,^[12]^ but this remains to be confirmed. In this case, both of the neoplasms were in advanced stages. The first aim of treatment was to relieve the patient's discomfort from the lower back pain, which was clearly due to the MM, and, thus, simple chemotherapy was used to alleviate the symptoms. Following this, surgery was performed to resect the lung cancer. Choosing the optimal chemotherapy agents for treatment was complicated by the presence of 2 cancers rather than just 1, and, thus, drugs were chosen that would treat both types of cancer. Bortezomib, a dipeptide proteasome inhibitor, is an antineoplastic agent approved for the treatment of MM and relapsed mantle-cell lymphoma that has been shown to be significantly cytotoxic against human nonsmall-cell lung cancer (NSCLC) cell lines in vitro and in a phase-I/II study.^[13,14]^

Bortezomib was used in conjunction with dexamethasone as the first-line therapy and the patient responded in a manner similar to MM patients without lung cancer. CR was achieved after four treatment cycles, and lenalidomide was used subsequently as a home maintenance therapy. Lenalidomide, an immunomodulatory drug, was originally approved to treat MM, but has also been reported to induce apoptosis in NSCLC cells.^[15,16]^ After 1 year of CR, the patient had a relapse of both cancers. Again, chemotherapy drugs were chosen that had been proven effective against both types of cancer: gemcitabine, vinorelbine, and dexamethasone. Using these therapeutics, PR was achieved and the patient was again given home maintenance therapy of lenalidomide. Since his diagnosis, the patient has survived for over 3 years and continues to be monitored for recurrence of the MM and/or lung cancer.

In summary, a patient presented with symptoms typical of MM. Using both comprehensive and targeted methods of examination, a diagnosis of MM with synchronous pulmonary adenocarcinoma was made. Prior to this case, only 1 other similar case has been reported in the literature. Due to the stage of lung cancer, surgery was used to resect the lung tumor. Following this, chemotherapy agents were chosen for treatment based on their ability to work against both types of cancer. Therefore, bortezomib was used as the first-line therapy and lenalidomide was used for at home maintenance. With this treatment plan, we have thus far achieved a satisfactory therapeutic effect as the patient is currently alive with both malignancies stable and nonprogressing.
